# Psychological wellbeing, physical impairments and rural aging in a developing country setting

**DOI:** 10.1186/1477-7525-7-66

**Published:** 2009-07-16

**Authors:** Melanie A Abas, Sureeporn Punpuing, Tawanchai Jirapramupitak, Kanchana Tangchonlatip, Morven Leese

**Affiliations:** 1 Health Service and Population Research Department, King's College London, London, UK; 2 Institute of Population and Social Research, Mahidol University, Nakhonpathom, Thailand; 3 Faculty of Medicine, Thammasat University, Pathumthani, Thailand

## Abstract

**Background:**

There has been very little research on wellbeing, physical impairments and disability in older people in developing countries.

**Methods:**

A community survey of 1147 older parents, one per household, aged sixty and over in rural Thailand. We used the Burvill scale of physical impairment, the Thai Psychological Wellbeing Scale and the brief WHO Disability Assessment Schedule. We rated received and perceived social support separately from children and from others and rated support to children. We used weighted analyses to take account of the sampling design.

**Results:**

Impairments due to arthritis, pain, paralysis, vision, stomach problems or breathing were all associated with lower wellbeing. After adjusting for disability, only impairment due to paralysis was independently associated with lowered wellbeing. The effect of having two or more impairments compared to none was associated with lowered wellbeing after adjusting for demographic factors and social support (adjusted difference -2.37 on the well-being scale with SD = 7.9, p < 0.001) but after adjusting for disability the coefficient fell and was non-significant. The parsimonious model for wellbeing included age, wealth, social support, disability and impairment due to paralysis (the effect of paralysis was -2.97, p = 0.001). In this Thai setting, received support from children and from others and perceived good support from and to children were all independently associated with greater wellbeing whereas actual support to children was associated with lower wellbeing. Low received support from children interacted with paralysis in being especially associated with low wellbeing.

**Conclusion:**

In this Thai setting, as found in western settings, most of the association between physical impairments and lower wellbeing is explained by disability. Disability is potentially mediating the association between impairment and low wellbeing. Received support may buffer the impact of some impairments on wellbeing in this setting. Giving actual support to children is associated with less wellbeing unless the support being given to children is perceived as good, perhaps reflecting parental obligation to support adult children in need. Improving community disability services for older people and optimizing received social support will be vital in rural areas in developing countries.

## Background

There is increasing interest worldwide in the study of wellbeing as a means to assess need and to evaluate positive dimensions of health care programs. Positive mental health "which allows individuals to realise their abilities, cope, and contribute to their communities" [1] and the capacity to sustain social relationships are key dimensions of wellbeing [2]. Wellbeing can be measured in terms of positive psychological symptoms (such as being able to enjoy things and to let go of worries) or life satisfaction, but increasingly multidimensional scales are used which include concepts such as autonomy, self-acceptance and relations with others [3,4].

Research on associations between physical impairments and wellbeing in older people has been limited [5-7] although there have been several studies of depression as an outcome suggesting that disability mediates most of the effect of specific medical conditions on depression [8-10]. However, research until now has come almost entirely from richer industrialised countries. One aim of this study was to see whether patterns of association between impairment, disability and psychological well-being in Thailand are similar to or different from those described elsewhere. Given cross-cultural differences in perceived well-being, a recent advance has been to develop culture-specific scales such as the Chinese Aging Well Profile (2007) [11]. In Thailand, Ingersoll-Dayton et al [12] developed and validated the Thai psychological well-being scale, which is related to the Scale of Psychological Well-being Scale [3]. Particular features of this, which is the only multidimensional wellbeing scale developed for use with Thai older people, is that compared to versions used in Western settings, more of the dimensions are interpersonal (measuring harmony and interconnectedness with other people) and fewer are intrapersonal (e.g. measuring acceptance and positive mood).

In Thailand, the setting for this study, the proportion of adults 60 years of age and over rose from 4.5% in 1960 to 9.5% in 2000 and is predicted to be 25% in 2040[13]. In the rural Thai context, as in many developing countries, facilities for health care and support for disabilities are limited. Also in many other developing countries, rapid rise in rural to urban migration of young adults means that older parents are increasingly living separately from their adult children [14]. In Thailand as in other Asian cultures, children traditionally take responsibility for older parents and older parents continue to support children. Given the potential relative importance of support from children [15] we were interested to see if support from children rather than support from others was associated with wellbeing.

## Methods

### Setting

We nested the study within the Kanchanaburi Demographic Surveillance System in western Thailand [16]. Kanchanaburi province is a mostly rural region located 130 kilometres west of Bangkok with a population of about 735,000 in 2007. The Kanchanaburi Demographic Surveillance System system has monitored households since 2000 in 100 neighborhoods (villages and urban census blocks). The neighborhoods were drawn from five strata (classified on ecological, socio-economic and population criteria) by stratified random sampling from the province population of 871 villages and 131 urban census blocks. The study described here is part of a longitudinal study designed to study the impact on older parents of out-migration of their adult children/offspring[17] During sampling for the main study we needed to identify which older adults were parents of at least one living child offspring, and whether the older parent was co-resident or not with at least one of their offspring. There was a potential sample of 3916 households with at least one older adult aged 60 and above, of whom 2432 (62%) had at least one child offspring of the older adult in the same household, and 1484 (38%) did not. We used simple random sampling to select 60% of households where an older adult was not co-resident with at least one of their child offspring and 30% of households where an older adult was co-resident with at least one of their child offspring. This comprised a total of 1620 households. We used random selection to identify the participant in situations where there was more than one eligible parent living in a household. Data were collected from November 2006 to Jan 2007.

### Recruitment

The interviewing team visited each sampling unit and made contact with the village headman prior to visiting each selected household. The populations were mostly already well acquainted with the demographic surveillance system. If the selected older adult and the household head gave consent, the interviewer first interviewed the household head with the household questionnaire and then the older adult with the individual questionnaire.

### Questionnaire development

We carried out focus group discussions to explore experiences of rural ageing, health and wellbeing and exchanges with family members. This informed the development of the questionnaire which was pre-tested by a team of ten experienced interviewers on three separate occasions. After each pre-test we made modifications by consensus. The final version was back-translated to English and checked for consistency by a bilingual psychiatrist and a bilingual social scientist.

### Inclusion criteria

Fluent Thai-speaking; aged 60 or over; parent of at least one living child (biological, adopted or step-child); residence in a demographic surveillance system village since at least 2004.

### Dependent variable

Psychological well-being. We used the 15-item Thai well-being scale [12,18], developed using extensive qualitative and quantitative methods. It has five dimensions of wellbeing which are harmony, interdependence with close persons, respect (from others), acceptance and enjoyment. Each dimension has three items which were developed from confirmatory factor analysis. We used the global factor model which was shown in Thailand to have good fit indices (goodness of fit 0.95, root mean square error of approximation 0.05) [12]. The items of the scale have been shown to have adequate internal consistency (Cronbach's alpha coefficient in this sample 0.89) and test-retest reliability (ranging from 0.6 to 0.7 in previous work) [12] and the scale correlated positively with life satisfaction and negatively with the Geriatric Depression Scale (-0.4) [12]. A statement is read out for each item. For example, for acceptance the statement is 'When you have small problems, you can let go of your worries'. The older person indicates on a 4-point scale if the statement is not at all true, slightly true, somewhat true or very true.

### Independent variable

Physical Illnesses and Impairments: we used a modified version of the Burvill physical illness scale [19]. Participants were asked about the presence of 13 common medical problems including breathlessness, faints/blackouts, arthritis, paralysis/loss of limb, skin disorders, hearing difficulties, heart trouble, eyesight problems, gastrointestinal problems, high blood pressure, diabetes and pain. If any of the problems was present we rated it as impairment if participants stated that the problem was interfering a great deal with their function.

### Potential confounders

#### Socio-economic position

years of education, number of household assets (out of 22, such as ownership of a fridge, motorcycle, or mobile phone), and household wealth index. We used principal components analysis to develop the household wealth index from the list of assets and the interviewer's global rating of household quality. The first principal component (which accounted for 26% of the variance compared to 7% for the second next most important) was used to provide an overall socioeconomic index based on these 23 items. This final index comprised 15 items (14 household assets plus household quality).

#### Social network and social support

We modified existing measures in the light of the importance in the Thai context of the family and of children. We measured size of neighbourhood family network, frequency of talking to a child, frequency of talking to friends, received support (instrumental, emotional, financial), actual support to children (instrumental, emotional, financial), perceived adequacy of support from and to children, and received support from others [20-22]. The received social support from children scale rated received support yes/no from any of their children on each of ten items. The received social support from others scale rated received support yes/no from anyone other than children on the same ten items. The support to children scale rated support to any children on each of five items.

#### Cognitive function

we used a learning task which has been used extensively in low and middle income countries which is drawn from the Consortium to Establish a Registry of Alzheimer's Disease (CERAD) [23,24], comprising immediate recall and delayed recall of a ten-word list. We defined significant cognitive impairment as performance at or below 1.5 standard deviations below the norm for the individual's age group and educational level on both tests.

#### Disability

We used the brief (12-item) questionnaire from the WHO Disability Assessment Schedule to rate disability over the past 30 days [25]. We were unable to translate the item on learning a new task, which was viewed as not applicable for older adults in this setting. Therefore, we used 11 items, each self-rated on a four point scale from no problem with carrying out the activity to total/extreme inability. Domains included understanding and communicating with the world, getting around, self-care, getting along with people, activities and participation in society. We categorised the total score into thirds of low, medium and high disability.

### Data collection

The data collection team of four supervisors and twelve interviewers had at least a bachelor's degree. Most had previous experience with interviewing for the demographic surveillance system. Residential training took ten days and included presentations, role play and practice in pilot villages. The study was presented to the interviewers as a study of healthy ageing in Thailand. Purposefully, no possible links were discussed between psychological wellbeing, impairment, disability or social support from children in order to blind the interviewers to the research hypotheses and none of these sections of the interview immediately followed each other in sequence.

The data collection team stayed in the villages at the headman's house or the temple. Quality control included checks on data completeness and consistency. Interviewers had to return to the participant if data were inadequate. Field station research managers (trained in the interview but blind to the hypothesis), and researchers were in frequent telephone contact and regularly visited the data collection teams. We conducted all interviews in Thai and gathered informed consent from all participants. We gained ethical approval from Kings College Research Ethics Committee (No. 05/05-68) and from Mahidol University Institutional Review Board.

### Sample size calculation

This was developed for the main longitudinal study which was designed to study the impact on older parents of out-migration of their adult children/offspring from the district [17]. The sample size was based on a comparison of prevalence of common mental disorder in those with all children migrated versus those with some children migrated and required a total sample size of 954 given the proportions expected of those exposed and not exposed to having all their children migrate from the district.

### Analysis

We used Stata version 9 for Windows (Release 9, College Station, TX: Stata Corporation. 2003). We weighted the data using the product of two sets of probability weights to take account of differential sampling at neighbourhood and household levels. The weighting at neighbourhood level took account of the probability of the neighbourhood being selected from the total number of neighbourhoods in that stratum in the province. The weighting at household level took account of the probability of being selected if the older parent was or was not co-resident with one of their offspring. We used the survey commands in Stata (svyset) for analyses. We first described the unadjusted associations between wellbeing score and the socio-economic, social support and health variables. We modelled impairment in two ways: as individual impairments and as a total of different impairments (one impairment versus none and two or more versus none). We used multiple linear regressions to develop a model for the effect of impairment on wellbeing, carrying out tests of the effect of impairment after adding in potential confounding variables. We explored interactions between social support, specific impairments, total impairments and total disability in the multivariable model. All tests were Wald tests as appropriate for weighted survey data. Residuals were computed for the final multivariable model and plotted as histograms (to assess any evidence for non normality, including individual outliers) and were also plotted against predicted values (to assess evidence for heteroscedasicity, in the sense of greater spread with increasing value). Variance inflation factors (VIFs) were computed for all independent variables to check for collinearity.

## Results

1620 older adults in 1620 households were sampled, of whom 1300 (80%) were eligible to take part. Reasons for not being eligible were having no biological or adopted children or step-children; having died since 2004, or moved out of the village. Out the 1300 eligible, 1147 (88%) agreed to take part and 153 (12%) were non -responders of whom 110 were unavailable for an interview (despite at least three visits to the household), 21 refused to take part and 22 were too unwell. Of the responders, data were incomplete for 43 due to the older adult being unwell or cognitively impaired. There were no significant differences between responders and non-responders in terms of age, gender, living alone, being married, or education.

### Demographic description of sample – Table 1

**Table 1:** Descriptive characteristics of parents: actual sample numbers (total n = 1147) and weighted percentages

	Study sample n = 1147	Weighted percentages
Female	n = 634	57%
Working	n = 564	48%
Marital status:		
Married	n = 633	54%
Widowed	n = 451	41%
Divorced/separated/single	n = 63	6%
Live alone	n = 155	9%
Education:		
None	n = 332	28%
1–3 years	n = 174	15%
Primary (4 yrs)	n = 541	49%
More than primary	n = 99	8%
Proportion with two or more limiting physical impairments	n = 540	50%
Cognitive impairment	n = 91	8%
At least one child living at home	n = 551	63%

Table 1 shows the actual sample numbers and weighted estimate of the characteristics in the wider province population of parents from which the sample was drawn. The average age was 70 years (SD 7.1). As shown in Table 1, 57% of the participants were female. Nearly half had less than primary school education, which for our sample meant less than four years education. (Only in the last two decades has Thailand's compulsory education extended to six and now to twelve years) Nearly half were still working. Because we over-sampled those not co-resident with a child, the study population has a lower proportion living with a child compared to the province estimate and is slightly more likely to live alone. Otherwise there were negligible differences between the study sample and the estimated province population. The average number of live children in these parents was 4.8 (SD 2.4); 2.4 sons and 2.4 daughters. Three-quarters either lived with a child or saw a child daily. The mean duration of residence in the same district was nearly 50 years. The mean wellbeing score was 33.3 (SD 7.6).

### Association between types of impairments and wellbeing – Table 2

**Table 2:** Prevalence of impairments and associations with wellbeing, weighted linear regression

Health impairments	Weighted percentages (95% confidence intervals)	Coefficient for association with wellbeing	P value for association with wellbeing	P value for association with wellbeing, adjusted for disability
Arthritis or rheumatism	44.4 (40.0–48.4)	-1.66	<0.001	0.915
Eyesight	23.3 (19.3–27.3)	-2.07	<0.001	0.202
Hearing	7.6 (6.0–9.2)	-.76	0.496	0.843
Cough	3.9 (2.4–5.4)	-2.89	0.110	0.306
Breathing	7.7 (5.4–10.0)	-2.73	0.024	0.186
High blood pressure	16.3 (13.0–19.5)	-0.48	0.415	0.185
Diabetes	7.1 (4.8–8.7)	-1.57	0.263	0.788
Heart trouble or angina	6.4 (4.1–8.7)	-1.12	0.534	0.831
Stomach or intestine	9.3 (6.6–12.0)	-2.50	0.008	0.086
Faints or blackouts	17.8 (14.5–20.9)	-2.63	0.001	0.143
Paralysis	2.3 (1.1–3.5)	-4.66	<0.001	0.012
Skin	3.4 (2.2–4.6)	0.02	0.993	0.785
Pain	37.3 (32.1–42.4)	-2.46	<0.001	0.105

The three most common impairments were arthritis, pain, and eyesight problems. Approximately one-third (32%) of the older adults did not have any impairment, 18% had one and 50% had two or more impairments. Impairments due to arthritis, pain, paralysis, vision, stomach problems or breathing were all associated with lowered wellbeing. Paralysis, faints/blackout, breathlessness, and pain were the impairments with the highest effect size for less wellbeing. After adjusting the impairments for disability, only paralysis remained significantly associated with low wellbeing.

### Association between number of impairments and wellbeing – Table 3

**Table 3:** Association between wellbeing score and having one or two or more physical impairments (sample n = 1147)

Number of physical impairments	Coefficient for having one impairment compared to none *	Coefficient for having two or more impairments compared to none *	Wald test F(2, 95)	*P *value
	-1.55	-3.03	15.52	<0.001
Adjusted for socio-demographic characteristics^1^	-1.01	-2.55	8.52	<0.001
Adjusted for ^1 ^+ social support and social network^2^	-0.64	-2.43	13.44	<0.001
Adjusted for ^1 ^+ ^2 ^+ social support to children^3^	-0.53	-2.37	14.42	<0.001
Adjusted for ^1 ^+ ^2 ^+ ^3 ^+ disability^4^	-0.23	-0.48	0.42	0.656
Adjusted for ^1 ^+ ^2 ^+ ^3 ^+ ^4 ^+ cognitive impairment^5^	-0.21	-0.46	0.38	0.685

As shown in Table 3, having one impairment compared to none and having two or more compared to none was significantly associated with less wellbeing. This association remained after adjusting for socio-demographic factors, social support from children, social support to children, and social support from others. There appeared to be some positive confounding by socio-demographic factors as the coefficients for the association with impairment fell slightly and the statistical significance decreased. This may be explained because factors such as wealth and education are associated with greater wellbeing and with less impairment. There appeared to be some slight negative confounding by social support from and to children as the significance rose again after adjusting for these. This could be because more impaired older peoples are likely to receive more social support from children and others, and more social support is also associated with greater wellbeing. Finally, after adjusting for disability, the association between number of impairments and wellbeing fell and was no longer significant.

### Multivariable model – Table 4

**Table 4:** Associations between psychological wellbeing and demographic, social and physical health status (sample n = 1147)

	Unadjusted Coefficient	Unadjusted P value	Adjusted coefficient*	Adjusted P value*
Older Age (years)	0.03	0.455	0.13	0.010
Female	-1.25	0.027	0.02	0.980
Currently working	0.43	0.419	1.32	0.018
Married versus widowed/single/divorced	1.05	0.066	0.33	0.581
Live alone	-1.06	0.113	-0.36	0.659
Education	1.48	0.007	0.15	0.145
Wealthy household	0.89	<0.001	0.32	<0.001
Physical impairment	-0.75	<0.001	-.37	0.020
Paralysis	-4.66	<0.001	-2.96	<0.001
Disability	-0.29	<0.001	-.22	<0.001
Cognitive impairment	-1.03	0.218	-1.43	0.223
Family social network size	0.11	<0.001	0.07	0.002
At least one child living in household versus no children in the household	-0.10	0.850	-0.57	0.304
Talk to a child at least weekly	0.93	0.002	0.74	0.029
Receiving support from children	0.51	<0.001	3.06	<0.001
Receiving financial remittances from children	2.18	<0.001	1.55	<0.001
Giving support to children	0.25	0.284	-0.62	<0.001
Receiving support from others	0.44	0.003	0.53	0.003
Perceive good support from children	3.79	<0.001	3.06	<0.001
Perceive giving good support to children	3.31	<0.001	1.26	0.029

* adjusted for all other variables in the table in a weighted regression.

Variables that were significantly associated with wellbeing either before and/or after adjustment are shown in Table 4. The parsimonious multivariable model for psychological wellbeing included age, household wealth, currently working, family network size close-by, receiving support from children, receiving support from others, talking more frequently to a child, perceiving receiving very adequate support from children, perceiving giving good support to children, less impairment due to paralysis, (p = 0.003), less general impairment, less disability, and giving less actual support to children. Of note, neither living alone or cognitive impairment were associated with wellbeing. The percentage of variance explained by the multivariable model was 32%. The residuals showed no evidence for non normality nor for outliers, and there was no evidence for heteroscedascity. There was no evidence for collinearity (all VIFs <10).

There was an interaction between social support from children and paralysis – those with low received social support from children and with paralysis were especially likely to have low wellbeing (p value for interaction 0.033).

## Discussion

The key finding from this paper is that impairment due to paralysis was associated with lowered psychological wellbeing in older Thai people, even after controlling for eleven other physical impairments, disability, socio-economic factors and social support. A second key finding is that while an increasing number of impairments was also associated with less wellbeing, this association, and those with other individual impairments, were explained by disability. A third finding is that in this Thai setting, received support from adult child offspring, received support from others and perceived support from adult child offspring were all independently associated with greater wellbeing in older parents whereas actual support to children was associated with lower wellbeing.

Chance is an unlikely explanation for the adjusted association between paralysis and low wellbeing, and for the adjusted association between disability and low wellbeing, as the associations were significant at a level of p = 0.001. We were able to adjust for a range of covariates so confounding is an unlikely explanation. All impairment and disability measures relied on subjective perception which may lead to misclassification of health status, although a high level of agreement has been reported between self-reported and objective health status measures [19]. Bias is unlikely in this community sample with a good response rate and interviewers were blind to the study hypotheses. Although we oversampled, this was on the basis on living arrangements rather than health and was anyway taken account of in the analysis. Non-systematic error is possible – for instance this might have come about through poor reliability of the interviewing team or through participants' errors in recall of their health problems, although previous work has shown a high level of agreement between self-report and objective health status measures [19]. We did not formally assess inter-rater reliability. However as part of the demographic surveillance system approach, quality control is well established and prioritised including daily checks on data completeness and consistency, having a research supervisor for each team of interviewers and having field station research managers (trained in the interview but blind to the hypothesis), and researchers, in frequent telephone contact and making regular visits to the data collection teams. This is a cross-sectional study so the direction of causality cannot be definitely inferred.

Why was paralysis associated with a large and significant effect on wellbeing? Studies of older people in Western countries have reported low mood and depression particularly following stroke and that this association was independent of disability [26]. Post-stroke depression of course may have a biological basis which may explain our finding [27]. However, wellbeing is a broader concept than depression. Our measure of wellbeing was developed and validated using thorough qualitative and quantitative work with Thai older people [12,18] and includes concepts vital to Thai wellbeing including interpersonal as well as intrapersonal aspects. The effect of paralysis may be due to the scarce disability services in rural Thailand, with few opportunities to receive aids, adaptations, or community transport. Rural people may thus be especially vulnerable to loss of social contacts in the neighbourhood and to losing respect. Another possibility is that impacts of stroke go beyond disability, either via biological effects on the brain [27] or through the psychological meaning of stroke such as shame over loss of function and altered appearance and fears about prognosis. In this setting of high out-migration, absence of children may also be a factor, although most older people still either live close to a child or talk to a child weekly or more.

Our finding that disability explains the association between number of impairments and low wellbeing echoes studies that have looked at impairment, disability and depression and at impairments and wellbeing in Western countries [6,9,28,29]. Prospective studies have shown that disability can predict the onset of depression [29]. A recent review concluded that much of the effect of impairment on negative affect could be explained by the potential mediating effect of disability [30]. It is striking that our result mirrors that from western countries, showing the cross-cultural applicability of the wellbeing model.

The model for greater wellbeing included other factors, notably received social support from children, perceived social support from children, received social support from others, financial remittances from children and wealth. As a number of associations were analysed in this study, a problem of multiple testing might have occurred. However, it is unlikely that this would explain our findings as most of the factors in the parsimonious model for wellbeing were significant at p < 0.001 or p = 0.001. Several possible mechanisms could explain the effect of received social support on wellbeing. Social support may reduce stress and consequently buffer the effect of negative events. Although received support is likely to reflect need, certain types of received support may be valuable in bringing about improved wellbeing[31].

Greater social support might also aid older people with impairment to carry out daily tasks, encourage them to be physically active, increase medication compliance, decrease social restriction and enhance self-esteem [32]. In the Thai culture, connections between parents and children are vital [33]. Although many parents in this study had out-migrant children, they continued to receive support through telephone contact, visits and economic remittances[17] In addition, they received support from others, often neighbours or other relatives living close by, and this was also independently associated with greater wellbeing. This suggests that older people living without children are adapting to the realities of out-migration and finding help from others close by in their neighbourhood. It is striking that received support from children and from others appeared helpful, and that received support from children may even buffer the impact of paralysis on low wellbeing. Older Thai people may place less value on autonomy than those in western countries, finding support from family members especially important and comforting [12]. A perception by the parent of giving a good amount of support to their offspring was associated with better well-being. However, giving actual support to children was associated with less wellbeing, perhaps reflecting parental obligation in this culture to support adult children in need [34].

Some limitations of this study include its cross-sectional design. Secondly our measure of wellbeing is culture specific – although this may also be regarded as strength of the study. Thirdly, the findings from this study might lack generalisability to all older adults as the sample was restricted to parents with at least one living child, although in Thailand this excluded only 5% of older people as we included anyone with a biological, adopted or stepchild.

In conclusion, disability may mediate most of the impact of chronic physical impairments on psychological wellbeing, although paralysis appears to have an independent effect. Received social support, perceived social support and wealth also have important positive effects on psychological wellbeing. Improving disability services and optimising social support will be vital in rural areas in developing countries which are likely to experience increasing depletion of younger adults in the next decade. While care is currently provided by family members, especially daughters and grand-daughters, we suggest that potentially valuable services in rural areas may include home care programmes for older people and their carers, home visits by health care volunteers in the village, day care, extending the existing network of 'elderly clubs', occupational therapy to enable aids and adaptations at home, and making a range of facilities more accessible to older disabled people,

## Conclusion

In conclusion, in this Thai rural setting, most of the association between physical impairments and lower wellbeing in older people is explained by disability. Received support from children and from others and perceived high support from and to children were all independently associated with greater wellbeing whereas giving actual support to children was associated with lower wellbeing. Improving community disability services for older people and optimizing received social support through families, neighbours and home care programs will be vital in rural areas in developing countries.

## Competing interests

The authors declare that they have no competing interests.

## Authors' contributions

All authors made substantial contributions to study design and interpretation of data. MA had main responsibility for analysing data and drafting the manuscript. SP and KT had main responsibility for acquisition of data. All authors were involved in revising the manuscript critically and have given final approval of the version to be published.

## References

[B1] World Health Organisation (2004). Promoting Mental Health; Concepts emerging evidence and practise, Summary report.Geneva.

[B2] World Health Organisation (2001). Strengthening mental health promotion. Geneva.

[B3] Ryff CD, Keyes CLM (1995). The structure of psychological well-being revisited. Journal of Personality and Social Psychology.

[B4] Tennant R, Hiller L, Fishwick R, Platt S, Joseph S, Weich S, Parkinson J, Secker J, Stewart-Brown S (2007). The Warwick-Edinburgh Mental Well-being Scale (WEMWBS): development and UK validation. Health Qual Life Outcomes.

[B5] Araki A, Murotani Y, Kamimiya F, Ito H (2004). Low Well-Being Is an Independent Predictor for Stroke in Elderly Patients with Diabetes Mellitus. Journal of the American Geriatrics Society.

[B6] Kendig H, Browning CJ, Young AE (2000). Impacts of illness and disability on the well-being of older people. Disabil Rehabil.

[B7] Smith J (2001). Well-being and health from age 70 to 100: findings from the Berlin Aging Study. European Review.

[B8] Beekman AT, Penninx BW, Deeg DJ, Ormel J, Braam AW, van Tilburg W (1997). Depression and physical health in later life: results from the Longitudinal Aging Study Amsterdam (LASA). J Affect Disord.

[B9] Ormel J, Kempen GI, Penninx BW, Brilman EI, Beekman AT, van Sonderen E (1997). Chronic medical conditions and mental health in older people: disability and psychosocial resources mediate specific mental health effects. Psychol Med.

[B10] Prince MJ, Harwood RH, Blizard RA, Thomas A, Mann AH (1997). Social support deficits, loneliness and life events as risk factors for depression in old age. The Gospel Oak Project VI. Psychol Med.

[B11] Ku PW, Fox K, McKenna J (2008). Assessing Subjective Well-being in Chinese Older Adults: The Chinese Aging Well Profile. Social Indicators Research.

[B12] Ingersoll-Dayton B, Saengtienchai C, Kespichayawattana J, Aungsuroch Y (2004). Measuring psychological well-being: insights from Thai elders. Gerontologist.

[B13] Skeldon R (1999). Migration of Women in the Context of Globalization in the Asian and Pacific Region. Women in Development Discussion Paper Series.

[B14] Deshingkar P (2006). Internal migration, poverty and development in Asia.

[B15] Chou KL, Chi I (2003). Reciprocal relationship between social support and depressive symptoms among Chinese elderly. Aging & Mental Health.

[B16] (2000). Report on baseline survey round 1.

[B17] Abas MA, Punpuing S, Jirapramukpitak T, Guest P, Tangchonlatip  K, Leese M, Prince M (2009). Rural-urban migration and depression in ageing family members left behind. British Journal of Psychiatry.

[B18] Ingersoll-Dayton B, Saengtienchai C, Kespichayawattana J, Aungsuroch Y (2001). Psychological well-being Asian style: the perspective of Thai elders. J Cross Cult Gerontol.

[B19] Burvill PW, Mowry B, Hall WD (1990). Quantification of physical illness in psychiatric research in the elderly. International Journal of Geriatric Psychiatry.

[B20] Wenger GC (1986). A longitudinal study of changes and adaptation in the support networks of Welsh elderly over 75. Journal of Cross-Cultural Gerontology.

[B21] Rindfuss RR, Jampaklay A, Entwisle B, Sawangdee Y, Faust K, Prasartkul P, Morris M (2004). The Collection and Analysis of Social Network Data in Nang Rong, Thailand. Network Epidemiology – A Handbook for Survey Design and Data Collection.

[B22] Sherbourne CD, Stewart AL (1991). The MOS social support survey. Soc Sci Med.

[B23] Ganguli M, Chandra V, Gilby JE, Ratcliff G, Sharma SD, Pandav R, Seaberg EC, Belle S (1996). Cognitive test performance in a community-based nondemented elderly sample in rural India: the Indo-U.S. Cross-National Dementia Epidemiology Study. International Psychogeriatrics.

[B24] Welsh KA, Butters N, Mohs RC, Beekly D, Edland S, Fillenbaum G, Heyman A (1994). The Consortium to Establish a Registry for Alzheimer's Disease (CERAD). Part V. A normative study of the neuropsychological battery. Neurology.

[B25] Epping-Jordan J, Ustun T (2000). The WHODAS-II: leveling the playing field for all disorders. WHO Mental Health Bulletin.

[B26] Stewart R, Prince M, Mann A, Richards M, Brayne C (2001). Stroke, vascular risk factors and depression: Cross-sectional study in a UK Caribbean-born population. Br J Psychiatry.

[B27] Kim JM, Stewart R, Kim SW, Yang SJ, Shin IS, Yoon JS (2006). Vascular risk factors and incident late-life depression in a Korean population. The British Journal of Psychiatry.

[B28] Braam AW, Prince MJ, Beekman AT, Delespaul P, Dewey ME, Geerlings SW, Kivela SL, Lawlor BA, Magnusson H, Meller I (2005). Physical health and depressive symptoms in older Europeans. Results from EURODEP. Br J Psychiatry.

[B29] Prince MJ, Harwood RH, Blizard RA, Thomas A, Mann AH (1997). Impairment, disability and handicap as risk factors for depression in old age. The Gospel Oak Project V. Psychol Med.

[B30] Prince M, Patel V, Saxena S, Maj M, Maselko J, Phillips MR, Rahman A (2007). No health without mental health. Lancet.

[B31] Boutin-Foster C (2005). Getting to the heart of social support: A qualitative analysis of the types of instrumental support that are most helpful in motivating cardiac risk factor modification. Heart & Lung: The Journal of Acute and Critical Care.

[B32] Greenglass E, Fiksenbaum L, Eaton J (2006). The relationship between coping, social support, functional disability and depression in the elderly. Anxiety, Stress and Coping.

[B33] Knodel J, Saengtienchai C, Sittitrai W (1995). The living arrangements of elderly in Thailand: views of the populace. Journal of Cross-Cultural Gerontology.

[B34] Zimmer Z, Korniek K, Knodel J, Chayovan N (2007). Support by migrants to their elderly parents in rural Cambodia and Thailand: A comparative study. Poverty, Gender and Youth Working Paper no 2.

